# Dietary Antioxidant Capacity Indices are Negatively Correlated to LDL-Oxidation in Adults

**DOI:** 10.1155/2023/5446163

**Published:** 2023-03-13

**Authors:** Mehran Nouri, Mitra Soltani, Milad Rajabzadeh-Dehkordi, Nastaran Rafieipour, Moein Askarpour, Maryam Najafi, Shiva Faghih

**Affiliations:** ^1^Department of Community Nutrition, School of Nutrition and Food Science, Shiraz University of Medical Sciences, Shiraz, Iran; ^2^Students' Research Committee, School of Nutrition and Food Science, Shiraz University of Medical Sciences, Shiraz, Iran; ^3^Health Policy Research Center, Institute of Health, Shiraz University of Medical Sciences, Shiraz, Iran; ^4^Department of Clinical Nutrition, School of Nutritional Sciences and Dietetics, Tehran University of Medical Sciences (TUMS), Tehran, Iran; ^5^Islamic Azad University, Rasht Branch, Rasht, Iran; ^6^Department of Clinical Nutrition, School of Nutrition and Food Science, Shiraz University of Medical Sciences, Shiraz, Iran; ^7^Nutrition Research Center, Shiraz University of Medical Sciences, Shiraz, Iran

## Abstract

**Introduction:**

Former research studies have demonstrated controversial associations between dietary indices and oxidative stress biomarkers including oxidized low-density lipoprotein (ox-LDL) and malondialdehyde (MDA). So, in this cross-sectional study, we aimed to assess the association of dietary total antioxidant capacity (DTAC), oxidative balance score, and phytochemical index (PI) with ox-LDL/MDA in a healthy adult population of Shiraz, Iran.

**Methods:**

236 individuals participated in this cross-sectional study. DTAC, OBS, and PI were calculated using a 168-item food frequency questionnaire (FFQ), which was previously validated in Iran. We measured ox-LDL and MDA in blood samples of the participants using commercially existing kits. Crude and adjusted models of linear regression were used to evaluate the relation of dietary indices with ox-LDL and MDA.

**Results:**

There was a significant association between ox-LDL and DTAC in both crude (*β* = −1.55; 95% CI: −2.53, −0.58; *P*-trend = 0.002) and adjusted (*β* = −1.65 95% CI: −2.66, −0.64; *P*-trend = 0.001) models. Also, a negative association was observed between ox-LDL and PI in the crude (*β* = −1.26 95% CI: −2.33, −0.29; *P*-trend = 0.01) and adjusted (*β* = −1.36 95% CI: −2.38, −0.34; *P*-trend = 0.01) models.

**Conclusion:**

Results of this study showed that DTAC and PI were inversely associated with ox-LDL as markers of lipid peroxidation. But no correlations were seen between MDA and dietary antioxidant indices.

## 1. Introduction

It is approved that oxidative stress play a key role in the pathogenesis of age-related chronic diseases such as diabetes mellitus, neurodegenerative diseases, cardiovascular diseases, cancers, chronic kidney disease, and chronic obstructive pulmonary disease [1]. The main carrier of cholesterol in the body is low-density lipoprotein (LDL). The protein and lipid content of LDL can go through oxidation, and as a result, accumulation of cholesterol occurs [2–4]. Moreover, this oxidized LDL (ox-DL) lead to platelet activating factor (PAF) and oxidized phospholipids (oxPLs) production by bringing changes in the activity of key enzymes [5, 6]. Similar to oxidized LDL (ox-LDL) another biomarker that can be widely assessed as a measurement of oxidative stress is malondialdehyde (MDA). MDA is an end product of lipid peroxidation of polyunsaturated fatty acids with cytotoxic and carcinogenic features [7, 8].

Endogenous and exogenous antioxidants protect body against free radicals and oxidative process [1]. Exclusively, dietary antioxidants which are known as exogenous antioxidants can activate antioxidant signaling, improve endogenous antioxidant status, and suppress oxidative stress [9]. Dietary total antioxidant capacity (DTAC) is an integrated measurement of dietary components that reflects the accumulative and synergistic interactions of foods [10]. It can be considered as a predictor of the total antioxidant status and the linkage between dietary antioxidants and oxidative stress in disease development [11]. Also, the oxidative balance score [12], which is referred as a balance between dietary and lifestyle pro- and anti-oxidants, contributes negatively with oxidative stress and related chronic diseases [13]. It is proved that the antioxidant components of OBS are involved in free radical scavenging specially those producing through lipid peroxidation, blocking pro-oxidant enzymes activities or expression, and eventually promoting activation of antioxidant enzymes [13, 14]. Phytochemicals are other natural compounds that bring about miscellaneous health benefits. Thus, dietary phytochemical index (PI) which is calculated by the percentage of calories derived from phytochemical-rich foods out of the total energy intake was introduced [15, 16].

It was previously reported that oxidative stress which is involved in the development of various chronic diseases can be influenced by dietary and lifestyle modifications. In addition, studies have illustrated that a combination of nutrients is more efficient than a single dietary factor in the risk of oxidative stress-related diseases due to their correlations [17, 18]. While some studies found significant correlation between dietary indices and oxidative biomarkers, some others have demonstrated controversial associations between dietary parameters and oxidative measurements including ox-LDL and MDA [19–22]. Also, an inverse relation was seen between DTAC and food pattern rich in antioxidants with inflammation markers [23, 24]. So, in this cross-sectional study, we aimed to assess the association of DTAC, OBS, and PI with ox-LDL/MDA in a healthy adult population of Shiraz, Iran.

## 2. Materials and Methods

### 2.1. Study Population

236 adults participated in this cross-sectional study. Study sample size was calculated based on *α* = 0.01, *β* = 0.10, and *r* = ±0.25 by the following formula:

(1) $$\begin{array}{c} C=0.5\times \ln\left[\frac{\left(1+r\right)}{\left(1-r\right)}\right]\text{,}\\ N=3+{\left[\left(Z_{1-\alpha /2}+Z_{1-\beta }\right)÷\;C\right]}^{2}\text{.} \end{array}$$

The age of the participants was between 20 and 50 years. We selected the participants from medical centers in Shiraz, Iran, by using cluster random sampling. We divided Shiraz into four sections and selected a community health center in each one. The participants did not have any chronic disease or did not follow any special diet (the details of this study have been previously published [25, 26]). This study was confirmed by the Shiraz University of Medical Sciences (IR.SUMS.REC.1394.S146).

### 2.2. Dietary Assessments

Participants' food intakes were evaluated using a 168-item food frequency questionnaire (FFQ), which was previously validated in Iran [27]. After extracting the food intakes in grams, NUTRITIONIST IV (version 7.0; N-Squared Computing, Salem, OR, USA) was used to calculate the intake of energy and nutrients.

Dietary TAC was acquired according to ferric reducing-antioxidant power (FRAP) [12]. This method measures the power of antioxidants of foods to convert ferric to ferrous ions [28]. The total mean of TAC was considered for similar foods (for example, some types of breads). At the end, the frequency of each food item intake was multiplied by its corresponding FRAP value, then summed up to estimate participant's DTAC [29, 30].

According to the method suggested by Goodman et al. [31], the OBS was calculated using the following items: intake of dietary pro-oxidants such as iron, polyunsaturated fatty acids (PUFAs), and saturated fatty acids (SFAs); nondietary pro-oxidants including smoking and obesity; dietary antioxidants such as fibers, folate, vitamin C, vitamin E, beta cryptoxanthin, lycopene, lutein/zeaxanthin, alpha-carotene, beta-carotene, selenium, and zinc; and nondietary antioxidants such as physical activity [32–35]. The scores of abovementioned 12 components were summed up and the OBS ranged between 0.0 and 24.0 (Table 1) [36, 37].

Dietary PI was computed using the method developed by McCarty [PI = (phytochemical-rich foods (g/d)/total food intake (g/d)) × 100] [16]. Food items included whole grains, nuts, legumes, olives, olive oil, soy products, also spices, coffee, and tea. Potatoes were often consumed as a starchy food and were not considered as vegetables. Natural juices of vegetables and fruits were included in the vegetable and fruit groups, because these components are also considered as high sources of phytochemicals [29, 30].

### 2.3. Other Assessments

5 cc blood sample was taken from each participant and stored at −70°C for ox-LDL and MDA assessment. Ox-LDL and MDA was measured using commercially existing kits (Pars Azmoon, Tehran, Iran). The participants' sex, age, alcohol use, and smoking habit were recorded through a face to face interview. Weight (with 100 g precision), height (with 0.5 cm precision), and waist circumference were measured and then BMI was calculated. Also, physical activity was evaluated by the International Physical Activity Questionnaire (IPAQ) [38].

### 2.4. Statistical Analysis

SPSS software (version 20.0, SPSS Inc., Chicago IL, USA) and STATA 16.0 were used for data analysis. The level of significance was *P* value <0.05. Crude and adjusted models of linear regression were used to evaluate the relation of dietary indices with ox-LDL and MDA. In adjusted models, the effects of age, energy intake, physical activity, BMI, sex, and smoking history were controlled.

## 3. Results

Table 2 illustrates the baseline characteristics of the participants. The mean of age, body mass index and waist circumference of the participants were 45.97 years, 28.28 kg/m^2^, 94.21 cm, respectively.

There was a significant association between ox-LDL and DTAC in both crude (*β* = −1.55; 95% CI: −2.53, −0.58; *P*-trend = 0.002) and adjusted models (*β* = −1.65 95% CI: −2.66, −0.64; *P*-trend = 0.001). Furthermore, a negative association was observed between ox-LDL and PI in the crude (*β* = −1.26 95% CI: −2.33, −0.29; *P*-trend = 0.01) and adjusted models (*β* = −1.36 95% CI: −2.38, −0.34; *P*-trend = 0.01). We observed no significant association between MDA and any of the dietary indices (DTAC, OBS, and PI) (Table 3).

## 4. Discussion

Based on the results of the present study, ox-LDL was inversely associated with DTAC and PI; however, no significant relationship was seen between OBS and ox-LDL. In addition, MDA was not related to the DTAC, OBS, or PI.

In line with our results, Hermsdorff et al. found a negative relationship between ox-LDL and DTAC in healthy young adults [19]. The key of similarity between these two studies findings was the study population. Indeed, both studies were conducted on healthy adults. Nevertheless, a 9-month observational study which included 35 postmenopausal women failed to find any significant correlation between DTAC and oxidative stress markers such as MDA, ox-LDL, and antioxidant enzymes [20]. In contrast, in a cross-sectional study conducted on 175 postmenopausal women, serum MDA levels were negatively associated with DTAC, while no relationship was seen between DTAC and ox-LDL [22]. The higher concentration of MDA in this study could be a potential reason for the inconsistency [22].

The results of the present study showed no correlation between OBS, MDA, and ox-LDL. We found no studies on the relationship between OBS, MDA, or ox-LDL. But in a study by Lakkur et al., F2-isoprostanes (FIP), which is an end product of MDA in lipid peroxidation, were negatively related to OBS [39]. Similarly, a case-control study on patients with colorectal cancer and the healthy control group showed the same negative association between OBS and FIP [40]. It should be mentioned that FIP is the gold standard biomarker of oxidative stress [41]. Considering that oxidative cascade includes different pathways with varied markers which are somehow linked to each other, the results of the other oxidative factors such as FIP can be suggestive of the probable correlations between OBS and MDA or ox-LDL. In a cross-sectional study of 206 participants, increased vitamin C and E consumptions were correlated with lower ox-LDL. Also, a similar relationship was reported between the higher fruits/vegetables intakes and reduced ox-LDL, which was faded after adjustment for saturated fatty acids, physical activity, and smoking (components of OBS) [42].

Regarding PI, in agreement with the result of the present study, adherence to Mediterranean diet resulted in lower ox-LDL levels in a cohort study. Moreover, fruits, vegetables, and olive oil intake were inversely associated with ox-LDL. As the Mediterranean diet includes a large proportion of whole grains, fruits, vegetables, and olive oils, it provides higher amounts of beta-carotene, vitamin B group, polyphenols, vitamin C, and vitamin E [43]. Apart from the observational studies, several intervention trials have shown that consumption of foodstuffs which exert antioxidant activities also resulted in plasma resistance to oxidation [44]. On the other hand, in a cross-sectional study on premenopausal women, greater PI was related to lower MDA levels [21]. Gender differences are particularly due to antioxidant features of estrogen; thus, less susceptibility of premenopausal women to oxidative stress might play a crucial role in the controversial results [45].

Antioxidants derived from diet neutralize exogenous or metabolic free radicals, prevent reactive species formations through inhibition of reactions with iron and copper, and prevent or repair susceptible structures including carbon-carbon bonds of PUFA, proteins, and DNAs [46, 47]. Furthermore, it has been illustrated that lipophilic antioxidants which are carried by LDL-C in the circulation system protect LDL-C from oxidation and as a result, suppressed ox-LDL concentrations [48].

Some limitations should be considered for this study. For instance, cross-sectional studies cannot clarify the exact effect of dietary intakes on oxidative biomarkers and vice versa, thus, clinical trials might be more efficient. In addition, while FFQ is a validated tool, its dependency on participants' memory might lead to under- or over-estimation of nutrients intakes. Moreover, since the present study was performed in Shiraz city, we should be cautious about extending the results to other regions. In addition, lower serum concentrations of oxidative markers in healthy populations might attenuate their associations with dietary components. It is worth noting that to the best of our knowledge, the current study is the first research evaluating the correlation between DTAC, OBS, and PI and MDA/ox-LDL.

Results of the present study expanded the findings of the modulatory effects of dietary anti- and pro-oxidants on the body's antioxidant defense system. In this cross-sectional study, while DTAC and PI were inversely associated with ox-LDL as a marker of lipid peroxidation, no correlations were seen between MDA and dietary antioxidant indices. Future studies are required to approve the positive influence of diets high in antioxidant on oxidation.

## Figures and Tables

**Table 1:** Oxidative balance score components.

OBS components	Assignment scheme
Nondietary pro-oxidants	
Obesity	0 = BMI ≥ 30 kg/m^2^ AND WC ≥ 1.02 m in males or ≥0.88 m in females 1 = BMI ≥ 30 kg/m^2^ OR WC ≥ 1.02 m in males or ≥0.88 m in females 2 = BMI < 30 kg/m^2^ AND WC < 1.02 m in males or <0.88 m in females
Smoking	0 = current, 1 = former, and 2 = never
Nondietary antioxidants	
Physical activity (MET-min/d)	0 = low (1^st^ tertile), 1 = medium (2^nd^ tertile), and 2 = high (3^rd^ tertile)
Dietary pro-oxidants	
SFAs (g)	0 = high (3^rd^ tertile), 1 = medium (2^nd^ tertile), and 2 = low (1^st^ tertile)
PUFAs (g)	0 = high (3^rd^ tertile), 1 = medium (2^nd^ tertile), and 2 = low (1^st^ tertile)
Iron (mg)	0 = high (3^rd^ tertile), 1 = medium (2^nd^ tertile), and 2 = low (1^st^ tertile)
Dietary antioxidants	
Vitamin E (mg)	0 = low (1^st^ tertile), 1 = medium (2^nd^ tertile), and 2 = high (3^rd^ tertile)
Vitamin C (mg)	0 = low (1^st^ tertile), 1 = medium (2^nd^ tertile), and 2 = high (3^rd^ tertile)
Provitamin A carotenoids (*µ*g)	0 = low (1^st^ tertile), 1 = medium (2^nd^ tertile), and 2 = high (3^rd^ tertile)
Lutein/Zeaxanthin (*µ*g)	0 = low (1^st^ tertile), 1 = medium (2^nd^ tertile), and 2 = high (3^rd^ tertile)
Lycopene (*µ*g)	0 = low (1^st^ tertile), 1 = medium (2^nd^ tertile), and 2 = high (3^rd^ tertile)
Selenium (*µ*g)	0 = low (1^st^ tertile), 1 = medium (2^nd^ tertile), and 2 = high (3^rd^ tertile)

OBS, oxidative balance score; BMI, body mass index; WC, waist circumference; MET, metabolic equivalent; SFA, saturated fatty acid; PUFA, poly-unsaturated fatty acid; *µ*g, microgram.

**Table 2:** Baseline characteristics of the study participants.

Variables	Mean ± SD or number (%)
Age (year)	45.97 ± 11.74
Weight (kg)	74.73 ± 14.30
Height (cm)	162.45 ± 9.65
BMI (kg/m^2^)	28.28 ± 4.69
Waist (cm)	94.21 ± 11.35
Hip (cm)	101.47 ± 9.64
WHR	0.90 ± 0.07
MDA (ng/L)	3.33 ± 0.62
ox-LDL (IU/L)	3.07 ± 3.14
Physical activity (Met.h/day)	21.09 ± 40.41
Energy (kcal/day)	2772.84 ± 1054.18
Carbohydrate (% energy)	61.03 ± 5.90
Protein (% energy)	12.74 ± 1.72
Fat (% energy)	26.22 ± 5.95
DTAC (mmol/100 g)	13.06 ± 5.45
OBS	12.91 ± 2.75
PI (% energy)	39.36 ± 19.57
Sex, male (%)	97 (41.1)
Marital status, married (%)	197 (84.9)
Education level, upper than high school (%)	171 (72.0)
Smoking history, no (%)	208 (88.1)

BMI, body mass index; WHR, waist to hip ratio; DTAC, dietary total antioxidant capacity; OBS, oxidative balance score; PI, phytochemical index. Values are mean ± SD for continuous and number (percentage) for categorical variables.

**Table 3:** Multivariate-adjusted model of the association of MDA and ox-LDL level with DTAC, OBS, and PI.

Variables	DTAC				OBS				PI			
	*T *_1_ (*n* = 78)	*T *_2_ (*n* = 79)	*T *_3_ (*n* = 79)	*P*	*T *_1_ (*n* = 104)	*T *_2_ (*n* = 63)	*T *_3_ (*n* = 69)	*P*	*T *_1_ (*n* = 79)	*T *_2_ (*n* = 78)	*T *_3_ (*n* = 79)	*P*
MDA (ng/L)												
Crude model	Reference	−0.04 (−0.24, 0.14)	−0.10 (−0.30, 0.08)	0.27	Reference	0.10 (−0.10, 0.30)	0.03 (−0.16, 0.23)	0.65	Reference	0.20 (0.006, 0.39)	−0.01 (−0.21, 0.17)	0.85
Adjusted model	Reference	−0.03 (−0.23, 0.16)	−0.10 (−0.30, 0.09)	0.30	Reference	0.03 (−0.17, 0.24)	0.01 (−0.19, 0.21)	0.88	Reference	0.20 (0.001, 0.40)	0.005 (−0.19, 0.20)	0.99
Ox-LDL (IU/L)												
Crude model	Reference	−1.20 (−2.17, −0.23)	−1.55 (−2.53, −0.58)	**0.002**	Reference	−0.29 (−1.30, 0.72)	0.57 (−0.41, 1.56)	0.31	Reference	−1.21 (−2.18, −0.23)	−1.26 (−2.23, −0.29)	**0.01**
Adjusted model	Reference	−1.21 (−2.22, −0.21)	−1.65 (−2.66, −0.64)	**0.001**	Reference	−0.38 (−1.44, 0.66)	0.65 (−0.38, 1.70)	0.28	Reference	−1.34 (−2.36, −0.32)	−1.36 (−2.38, −0.34)	**0.01**

MDA, malondialdehyde; ox-LDL, oxidized low-density lipoprotein; DTAC, dietary total antioxidant capacity; OBS, oxidative balance score; PI, phytochemical index. Adjusted for age, energy intake, BMI, sex, physical activity, and smoking history. Obtained by linear regression. Significant values are shown in bold.

## Data Availability

The data used to support the findings of this study are available on request from the authors.
